# Drug-Loaded, Polyurethane Coated Nitinol Stents for the Controlled Release of Docetaxel for the Treatment of Oesophageal Cancer

**DOI:** 10.3390/ph14040311

**Published:** 2021-04-01

**Authors:** Paris Fouladian, Qiuyang Jin, Mohammad Arafat, Yunmei Song, Xiuli Guo, Anton Blencowe, Sanjay Garg

**Affiliations:** 1 Pharmaceutical Innovation and Development (PIDG) Group, Clinical and Health Sciences, University of South Australia, Adelaide, SA 5000, Australia; paris.fouladian@mymail.unisa.edu.au (P.F.); mohammad.arafat@mymail.unisa.edu.au (M.A.); May.Song@unisa.edu.au (Y.S.); 2 Key Laboratory of Chemical Biology (Ministry of Education), Department of Pharmacology, School of Pharmaceutical Sciences, Shandong University, Jinan 250012, China; jcas09@163.com; 3 Applied Chemistry and Translational Biomaterials (ACTB) Group, Clinical and Health Sciences, University of South Australia, Adelaide, SA 5000, Australia

**Keywords:** drug-eluting stent, self-expandable metal stent, controlled release, docetaxel, oesophageal cancer

## Abstract

For several decades, self-expanding metal stents (SEMSs) have shown significant clinical success in the palliation of obstructive metastatic oesophageal cancer. However, these conventional oesophageal stents can suffer from stent blockage caused by malignant tumour cell growth. To overcome this challenge, there is growing interest in drug-releasing stents that, in addition to palliation, provide a sustained and localized release of anticancer drugs to minimise tumour growth. Therefore, in this study we prepared and evaluated an oesophageal stent-based drug delivery platform to provide the sustained release of docetaxel (DTX) for the treatment of oesophageal cancer-related obstructions. The DTX-loaded oesophageal stents were fabricated via dip-coating of bare nitinol stents with DTX-polyurethane (PU) solutions to provide PU coated stents with DTX loadings of 1.92 and 2.79% *w*/*w*. Mechanical testing of the DTX-PU coated stents revealed that an increase in the drug loading resulted in a reduction in the ultimate tensile strength, toughness and Young’s modulus. In vitro release studies showed a sustained release of DTX, with ~80–90% released over a period of 33 days. While the DTX-loaded stents exhibited good stability to gamma radiation sterilisation, UV sterilisation or accelerated storage at elevated temperatures (40 °C) resulted in significant DTX degradation. Cell proliferation, apoptosis and Western blotting assays revealed that the DTX released from the stents had comparable anticancer activity to pure DTX against oesophageal cancer cells (KYSE-30). This research demonstrates that the dip-coating technique can be considered as a promising approach for the fabrication of drug-eluting stents (DESs) for oesophageal cancer treatment.

## 1. Introduction

Oesophageal cancer is the seventh most common cancer in men and the thirteenth most common cancer in women, with more than 500,000 new cases reported in 2018 [1]. Oesophageal cancer has two distinct histological subtypes, which carry varying risk factors, contributing to the widespread incidence rates of the disease [2]. The vast majority of cases are related to squamous cell carcinoma, with has a high incidence rate in developing countries [3]. However, the rate of adenocarcinomas is dramatically increasing with a greater burden in high-income countries [4]. Depending on the stage of the disease, several treatments are currently used including surgery, chemotherapy, radiotherapy, electrocoagulation and laser therapy [5]. However, oesophageal cancer is often challenging to treat and only patients diagnosed in the early stages of the disease have high survival rates [6]. The most prominent symptom associated with oesophageal cancers is dysphagia, which causes difficulties in swallowing foods and drinking. Surgery, as the first-line treatment option, is only effective in 50% of patients [6,7] and oesophageal stenting is a widespread palliation approach commonly used for relieving dysphagia in the remaining patients. Self-expandable metal stents (SEMS) are a very common type of stent used for relieving dysphagia and have been shown to be effective in improving patients’ quality-of-life [8].

The benefit of the conventional gastrointestinal stents is limited in many cases due to lumen reocclusion that often occurs as a result of tumour ingrowth and overgrowth [9,10,11,12]. The incorporation of drug substances within stent platforms has resulted in a new generation of stents called drug-eluting stents (DESs) [13,14]. DESs provide dual functions, including mechanical support of the lumen and localized, sustained release of therapeutic agents, and can be considered as a potential solution to restenosis-related issues [15]. Controlled drug elution is often achieved by the use of a drug-impregnated polymer membrane or coating that acts as a drug reservoir that can sustain the drug release [16].

Several approaches and drug-polymer combinations have been employed to prepare DESs for the delivery of anticancer drugs for the treatment of oesophageal cancers [5,17,18,19,20]. Liu et al. prepared drug-loaded (5-fluorouracil or paclitaxel) poly(ethylene-co-vinyl acetate) (PEVA) films via melt processing, which were then rolled around the outer surface of the SEMS [5]. The evaluation of their performance in vivo in porcine revealed localized drug accumulation in the oesophageal lumen relative to other organs, although stent migration was noted as a limitation. Similarly, in many other studies directed towards the development of DESs, drug-loaded polymeric films have been prepared which are then rolled around the stent, or only the films are investigated [9,15,17]. While these film covered stents have shown promise, they tend to suffer from migration [5], and in some cases, tumour in-growth has resulted in the fracturing of the film and the blockage of the lumen [21].

The application of other coating approaches to prepare DES, such as dip-coating, has been limited mainly to the preparation of drug-eluting coronary stents [22,23,24]. For example, Heldman et al. prepared paclitaxel drug-eluting coronary stents via dip-coating and showed in vivo efficacy in a porcine model for the prevention and treatment of coronary restenosis [25]. Acharya et al. employed dip-coating to prepare stents coated with poly(caprolactone) encapsulating a nitric oxide prodrug, *S*-nitrosoglutathione [26]. Multiple dip-coating cycles were implemented to prepare multiple-layer coatings, with an increase in the layers correlating to an increase in the burst release, although the overall drug release plateaued at ~70–80% by day 11 regardless of the number of layers. Whilst these studies have been performed on coronary DESs, no investigations employing dip-coating have been reported for the fabrication of oesophageal stents, although we have recently demonstrated the potential of this approach for the preparation of 5-fluorouracil-loaded drug-eluting colonic stents [27].

Docetaxel (DTX) is a semisynthetic taxoid derivative with a high potency against human oesophageal cancers [17]. DTX is a cell growth inhibitor and acts on stabilizing microtubules through inhibition of cell mitosis [17,28]. Currently, Taxotere is the only commercial intravenous formulation of DTX that rapidly reaches high plasma concentrations leading to severe systemic side effects such as neutropenia, musculoskeletal toxicity and neuropathy [29,30]. Furthermore, the rapid clearing of DTX before reaching therapeutic concentrations in oesophageal tumours can limit the effectiveness of the treatment. Thus, localized delivery systems could significantly improve the efficacy of DTX therapies for the treatment of oesophageal cancers.

To combine the mechanical support of oesophageal stents and the longer-term benefit of localized, sustained release of chemotherapeutics, we aimed to prepare and evaluate an oesophageal stent-based drug delivery platform to provide the sustained release of DTX for the treatment of oesophageal cancer. The DTX-loaded oesophageal stent was developed using dip-coating. Chronosil™ 80A, a polycarbonate-based aliphatic polyurethane with 5% silicone was used as the matrix material. Previously, we have demonstrated that Chronosil is an excellent polymer matrix for the fabrication of drug-eluting films and coatings, in particular, due to its near-linear release of DTX over 30 days [29]. A variety of properties of the fabricated stents, including mechanical properties, drug release behaviour, stability after sterilization, and short-term accelerated stability were investigated. In addition, the cytotoxicity of DTX released from the stents was tested in vitro against an oesophageal squamous carcinoma cell line (KYSE-30) using MTT, Hoechst and Western blotting assays.

## 2. Results and Discussion

### 2.1. Preparation of Dip-Coated DTX-Loaded Stents

DTX-loaded stents with two different DTX-loadings (1.92 and 2.79% *w*/*w* relative to the polyurethane (PU) coating) were prepared via dip-coating with DTX/PU (Chronosil) solutions. Bare nitinol stents were immersed in DTX/PU solutions, mechanically withdrawn at 6.5 mm/s and air-dried for 10 min before repeating the process to obtain thicker coatings. Finally, the stents were dried at 40 °C for two days to obtain the DTX-loaded stents. The total mass of the DTX/PU coatings for the 1.92% and 2.79% *w*/*w* drug loaded stents were 559.47 ± 8.42 and 563.93 ± 15.70 mg/stent, respectively. Multiple stents (*n* = 8) were dip-coated with excellent reproducibility as indicated by the low standard deviation of the deposited coating mass. Deposition of the coatings on the metal stents resulted in negligible changes in the stent dimensions (Supplementary Information (SI), Table S1). In addition, drug-free PU coated stents were prepared using the same dip-coating procedure.

### 2.2. Characterisation of DTX-Loaded Stents

Previously, our group conducted a comprehensive characterisation of DTX/PU (Chronosil) films using Fourier transform infrared spectroscopy (FTIR), X-ray diffraction (XRD), differential scanning calorimetry (DSC) and thermal gravimetric analysis (TGA) [29]. Therefore, in this study we focused on characterising the morphological features and mechanical properties of the DTX-loaded stents, the DTX release and stability, and the cytotoxicity of the released DTX.

**Figure 1:**
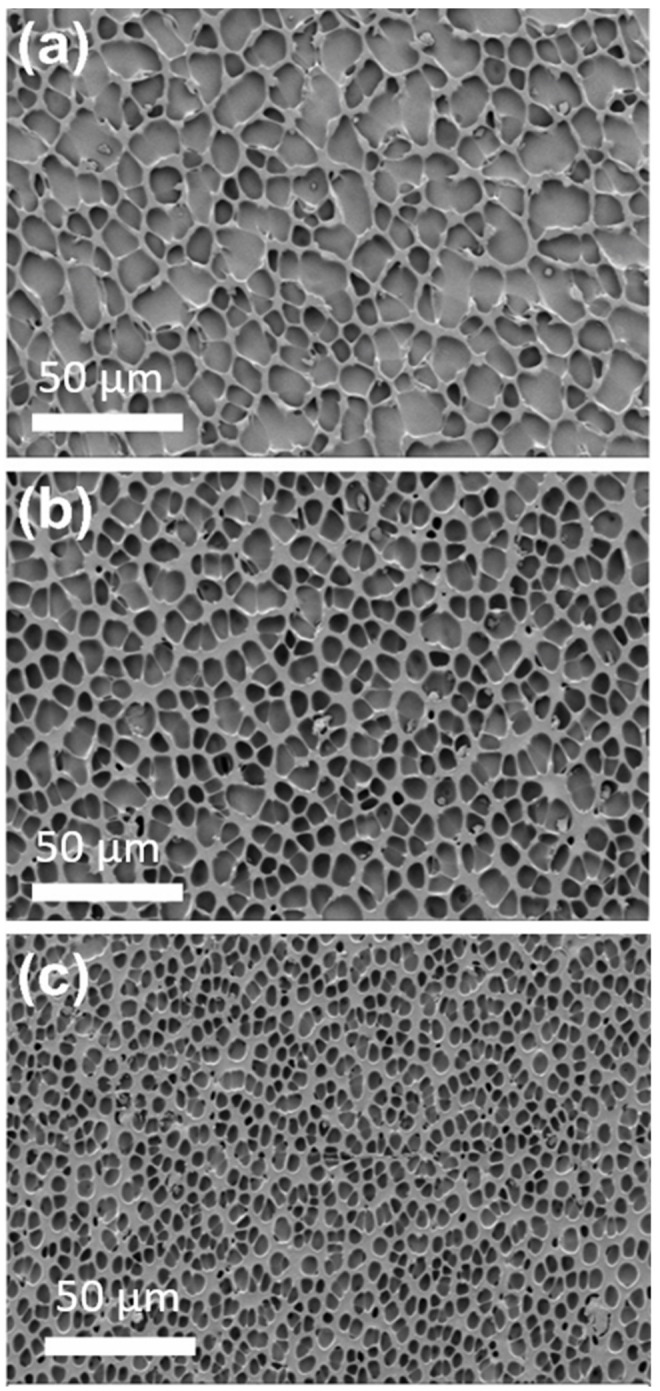
Scanning electron microscopy (SEM) images of (**a**) drug-free PU, (**b**) 1.92% *w*/*w* DTX-loaded and (**c**) 2.79% *w*/*w* DTX-loaded stents. The cavity sizes for the drug-free, 1.92 and 2.79% *w*/*w* DTX-loaded stents sections were 12.6 ± 4.6, 8.4 ± 2.1 and 4.9 ± 1.6 μm (*n* = 50), respectively.

The surface morphology of the DTX-loaded stents (1.92 and 2.79% *w*/*w*) was characterized via SEM (Figure 1) and compared to drug-free PU coated stents. The drug-free stent coatings presented with irregular polygonal cavities with sharp and narrow ridges on a flat sublayer. In contrast, the 1.92 and 2.79% *w*/*w* DTX-loaded stents displayed similar cavities with smaller sizes and wider ridges, and this structural change appeared to correlate with the DTX loading. The observed features are believed to arise during solvent evaporation resulting in water condensation as the surface cools, leading to a breath figures-type process and the formation of ill-defined honeycomb structures [31,32,33]. Similar polygonal features have previously been noted for PU honeycomb films [34]. In particular, García Fernández-Luna et al. reported the effect of small molecule hydrophobic additives (isatin thiosemicarbazone derivatives) on structure of PU and poly(methyl methacrylate) honeycomb films, which displayed structural changes in response to changes in concentration and type of additive [35]. Therefore, the structural changes observed for the DTX/PU coatings with increasing DTX concentration are believed to arise from modification of the interfacial properties of the PU. Conceivably, changes in the surface structure can affect the surface area, which may contribute to variability in DTX release rates, with larger surface areas resulting in faster drug release.

**Figure 2:**
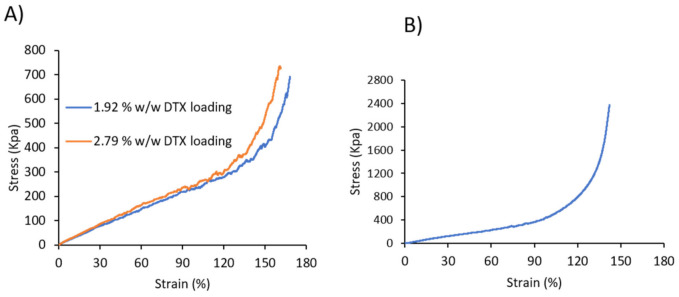
Stress–strain curve of (**A**) DTX-loaded 3D printed and (**B**) commercial stents.

The mechanical properties of the DTX-loaded and commercial coated stents were investigated through tensile testing until the stents’ diameter reduced to 8 mm (stent introducer diameter), and the ultimate tensile strength, elongation at 8 mm diameter, toughness and Young’s Modulus were determined (Figure 2 and Table 1). Comparing the drug-loaded and commercial stents, it was evident that the commercial stent possessed a higher ultimate tensile strength, toughness and Young’s modulus compared to the drug-loaded stents, which is due to the differences in the coating material (type of polymer), coating thickness, metal mesh thickness and design of the stents. Nevertheless, the lower ultimate tensile strength, toughness and Young’s modulus recorded for the DTX-loaded stents indicates that less force is required to collapse the stents, which may be beneficial for their insertion and deployment from stent introducers that are used to position stents in the oesophagus. For the DTX-loaded stents, there was a slight reduction in all parameters with an increase in drug-loading, which is hypothesized to result from DTX interfering with the H-bonding interactions between the urethane segments of the PU, as noted in our previous study [29]. The flexibility and recoverability of the DTX-loaded stents were evaluated using a commercial stent introducer (SI, Figure S1). The stents were compressed into the catheter and released >10 times, and instantaneously recovered back to their original dimensions with no apparent peeling or fracture of the coatings observed.

### 2.3. In Vitro Release of DTX from Drug-Loaded Stents

**Figure 3:**
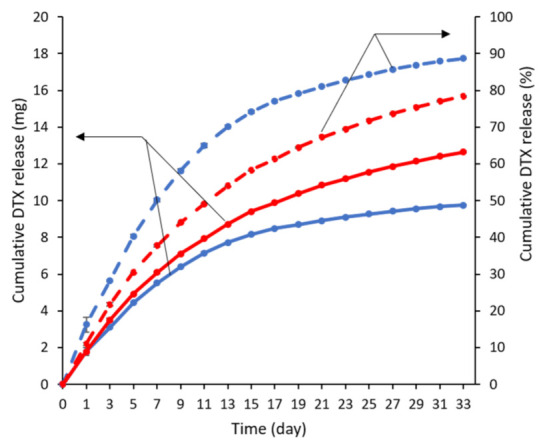
In vitro release profiles of DTX from 1.92 (blue) and 2.79% *w*/*w* (red) DTX-loaded stents over 33 d, conducted in PBS at pH 6.5 and containing 0.1% *v*/*v* Tween 80 (*n* = 3). Solid and dashed lines represent the cumulative DTX release in mg and percentage, respectively. Error bars are smaller than symbols.

The release of DTX from the stents was carried out in vitro by submerging the stents in PBS containing 0.1% *v*/*v* Tween 80. The oesophageal environment is slightly acidic (between pH 4 and 7) [36], and therefore, a pH of 6.5 was employed. The amount of the drug released was determined using HPLC for a period of 33 d (Figure 3), and the release media were completely changed every two days to maintain sink conditions. DTX release from the stents was faster over the first seven days and followed by a period of slower release for both DTX loadings. In the first seven days, almost half of the drug was released in the 1.92% *w*/*w* DTX-loaded stent while ~37% was released from the 2.79% *w*/*w* DTX-loaded stent. The faster release in the first several days may be useful in reducing the tumour burden while the sustained release will suppress any growth from the remnant cancer cells [29]. The total amount of DTX released from the 1.92 and 2.79% *w*/*w* drug-loaded stents over 33 d was determined to be 89 and 78%, respectively.

### 2.4. DTX-Loaded Stent Stability Studies

#### 2.4.1. Accelerated Stability Studies

To assess the stability of DTX in the drug-loaded stents, stent pieces were sealed in foil bags and stored under ambient conditions (25 °C), 25 °C/60% RH and 40 °C/75% RH, and the DTX content was determined via HPLC over three months and compared to the initial amount (Table 2). For the stent pieces stored under ambient and 25 °C/60% RH conditions, the percentage of DTX remaining was >90% over the first two months. In comparison, the stent pieces stored at 40 °C/75% RH showed more significant reductions with a decrease of DTX of ~18–27% after three months, respectively. Overall, these results imply that the DTX in the stents is reasonably stable at room temperature (25 °C), but higher temperatures (40 °C) cause the accelerated degradation of the DTX.

#### 2.4.2. Stability of DTX in Stents to Gamma and Ultraviolet (UVC) Irradiation

Stent pieces from the 1.92% *w*/*w* drug-loaded stents were subjected to sterilisation using gamma (25 kGy) and UV (λ = 254 nm, 15 W, 20 min) irradiation, and the amount of DTX present was determined before and after sterilisation. Exposure to gamma and UV irradiation resulted in a ~8 and 36% reduction in DTX, respectively, (Table 3) which is consistent with results reported previously by Shaikh et al. [17]. Although DTX is a photosensitive drug [37], its inclusion within a polymer matrix might be expected to improve its stability [17]. Nevertheless, the incorporation of the drug into the PU matrix did not appear to improve the stability of the drug to UV irradiation. Conversely, gamma irradiation only led to a slight degradation of DTX in the stents. Thus, it can be concluded that gamma irradiation would be preferable for the sterilization of DTX-loaded stents, with negligible reduction of the DTX content.

### 2.5. In Vitro Assessment of DTX-Loaded Stents

#### 2.5.1. MTT Assay

**Figure 4:**
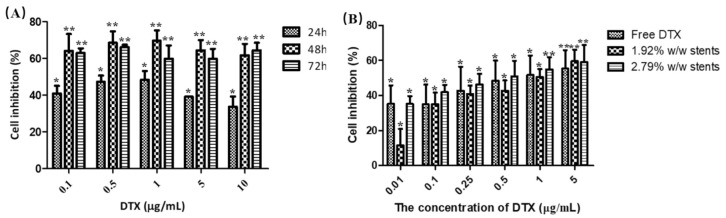
Cell inhibition caused by (**A**) various concentrations of pure DTX incubated with human oesophageal cancer cells (KYSE30) for 24, 48 and 72 h, and (**B**) various concentrations of pure DTX and DTX released from the stents incubated with KYSE30 cells for 48 h, as measured using a MTT assay (**B**). Data are presented as the mean ± SD of at least three independent experiments. * *p* < 0.05, ** *p* < 0.01 vs. untreated group. (*n* = 3).

The cytotoxicity of pure DTX after 24, 48 and 72 h were assessed against KYSE30 cells using the MTT assay (Figure 4A). The diluted DTX drug concentrations ranging from 0.1 to 10 µg/mL showed a dose-dependent reduction in the viability of KYSE30 cells and induced the maximum inhibition of cell viability at the concentration of ~0.1–1 μg/mL after 48 h treatment. The IC_50_ value for pure DTX was found to be 1.95 ± 1.28 µg/mL. To evaluate the cytotoxicity of the DTX released from the stents (1.92 and 2.79% *w*/*w* drug loadings), an MTT assay was conducted against KYSE30 cells after incubation for 48 h (Figure 4B). The results showed that two stents exhibited similar inhibitory effects to pure DTX with the IC50 values at 1.00 ± 0.15 µg/mL (1.92% *w*/*w* stent) and 0.81 ± 0.13 µg/mL (2.79% *w*/*w* stent), respectively. These results demonstrate that the DTX released from the stents induced an obvious inhibitory effect on the proliferation of KYSE30 cells, indicating that the DES, as a controlled-releasing system, possessed similar antitumour effects to the pure DTX in KYSE30 cells.

#### 2.5.2. Hoechst Assay

**Figure 5:**
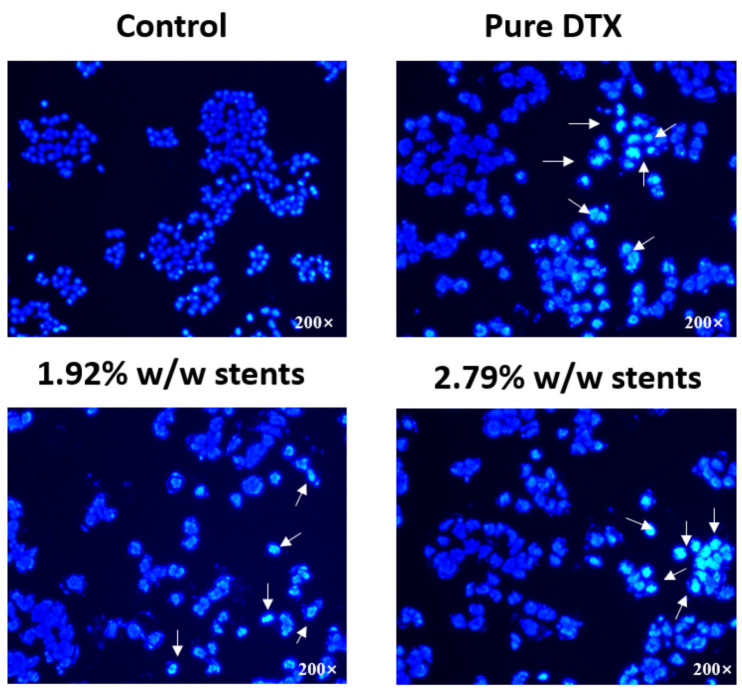
Fluorescence microscopy images showing the morphological differences of the nuclei of human oesophageal cancer cells (KYSE30) after incubation with DTX released from the stents and pure DTX (1 µg/mL for all) for 48 h. Cells were fixed and stained with DNA-binding fluorochrome Hoechst 33,342 before visualisation. Arrows indicate characteristic apoptotic cells. (*n* = 3).

Most chemotherapy drugs kill tumour cells through the induction of apoptosis, which is distinguished by a number of characteristic morphological changes in the cellular structure, together with a number of enzyme-dependent biochemical processes [38]. Some studies have shown that DTX induced apoptosis in lung cancer, oesophageal cancer, ovarian cancer and breast cancer [39,40,41]. It is reported that DTX inhibits cell division by stopping cell proliferation through stabilizing microtubules, and it initiates apoptosis by binding to β-microtubules and thus blocking mitosis [42]. Thus, we firstly observed the morphological changes of KYSE30 cell nuclei after treatment with DTX released from the stents and pure DTX using the Hoechst 33,342 staining assay (Figure 5). The results showed an increased number of cells with characteristics of apoptosis, such as reduced nuclear size, chromatin condensation and nuclear fragmentation in the three treatment groups compared with control group, indicating that DTX released from stents induced the apoptosis of KYSE30 cell.

#### 2.5.3. Apoptosis Assay

**Figure 6:**
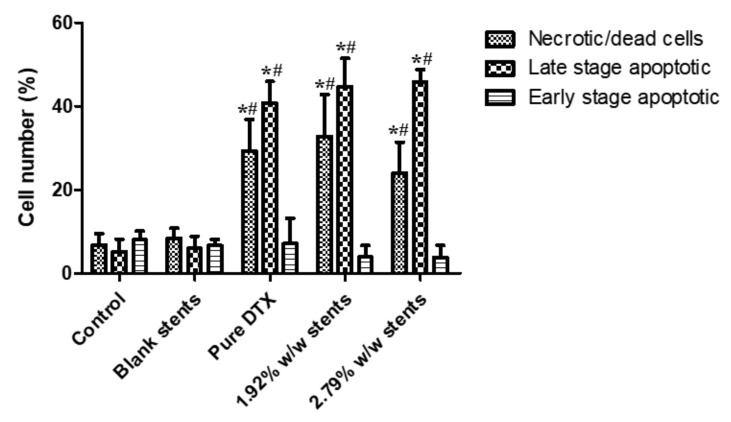
Apoptosis progression in KYSE30 esophageal cancer cells after treatment with blank (drug-free) stent, pure DTX drug (1 µg/mL) and DTX-loaded stent samples for 48 h, as analysed by flow cytometry-based annexin V-FITC/PI double staining. Data are presented as the mean ± SD of at least three separate experiments. * *p* < 0.05 vs. control group. # *p* < 0.05 vs. blank stents. (*n* = 3).

Annexin V-FITC/PI double staining assay was then performed to assess the proapoptotic activity of DTX released from the stents and pure DTX (1 µg/mL for all) incubated with KYSE30 cells for 48 h. As shown in Figure S2, the rate of Annexin V-positive revealed that apoptotic cells were significantly increased by treating the cell line with pure DTX solution, 1.92 and 2.79% *w*/*w* drug eluting stents leachables compared to drug-free stents leachables (blank stents) and control group, which illustrated that DTX induced a notable level of apoptosis. As illustrated in Figure 6, pure DTX solution and DTX released from DES induced significant increases in late stage apoptotic cells, and the total apoptosis rates were increased dramatically compared to the control group and blank stents group. Likewise, the number of necrotic/dead cells were also increased after treatment with pure DTX solution and DESs leachables. Meanwhile, there was no significant difference between the blank stent and the control group, indicating that drug free stents do not have an obvious effect on the apoptosis of KYSE30 cells. These results, along with the morphological changes observed by the Hoechst 33,342 assay, demonstrated that DTX could inhibit the development of oesophageal cancer cells by inducing apoptosis. Necrosis has been considered to be an accidental, unregulated type of cell death that is triggered by various factors external to the cells or tissues, such as infection, toxins and ROS, and has been demonstrated to be involved in some drug-induced tumour cell death [43,44]. As shown in Figure 6, the number of necrotic/dead cells were also increased after treatment with pure DTX solution and DESs leachables, indicating that DTX could induce the necrosis of KYSE30 cells and thereby inhibit their development. A previous study reported that DTX could show an antitumour effect through inducing cell cycle arrest at the G2/M phase [39]. As reported, the blockage of cell cycle progression usually leads to cell apoptosis, which is the programmed cell death governing the cell survival/death balance, indicating that DTX induced apoptosis after cell cycle arrest.

#### 2.5.4. Western Blotting Assay

**Figure 7:**
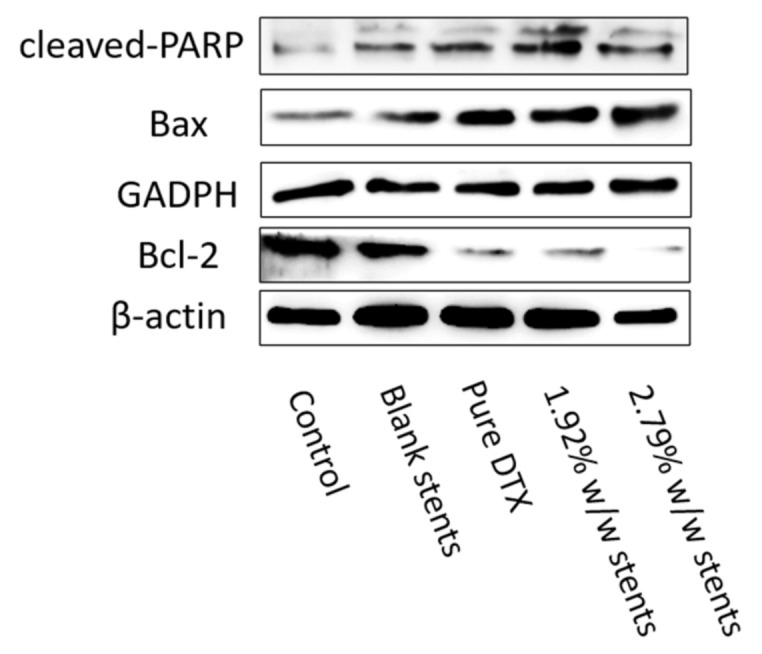
DESs regulated the expressions of apoptosis-related proteins. Pure DTX and DESs promoted the expression of cleaved-PARP and Bax, while inhibited the expression of Bcl-2. The expression of cleaved-PARP, Bcl-2 and Bax in KYSE30 after treatment with DESs and pure DTX were detected by Western blotting assay. Both GAPDH and β-actin were used as loading control.

Most apoptosis in vertebrates occurs through a mitochondria-dependent pathway that is mainly mediated by proapoptotic proteins Bax from the Bcl-2 family. This pathway leads to a mitochondrial permeability transition by inhibiting Bcl-2 [45]. PARP represents a protein family that plays an important role in many cellular processes, such as DNA repair, genomic stability, and programmed cell death, and is considered to be a biomarker for the detection of apoptosis [46]. The cleavage of this protein is associated with the dramatic morphological alterations in all apoptosis cell death cases [46]. Accumulating evidence has demonstrated that apoptosis induced by taxane group chemotherapeutic agents involves several apoptotic signal molecules containing JNK, p53/p21, Ras/Bcl-2, MAPKs, etc. [47]. Thus, to further study the effect of DTX released from the stents and pure DTX on KYSE30 cells, the activation of PARP, as well as the expression of Bcl-2 family proteins such as antiapoptotic Bcl-2 and the pro-apoptotic protein Bax, was examined. The increased cleaved-PARP and Bax, coupled with decreased Bcl-2, indicated that DTX released from the stents, induced a notable level of apoptosis in KYSE30 cells via the mitochondrial apoptosis pathway (Figure 7).

## 3. Materials and Methods

### 3.1. Materials

The bare flared-shaped nitinol oesophageal stents (60 mm length, 30 mm diameter at the two flared ends and 28 mm throughout the body; Micro-Tech, Nanjing, China) were purchased from CK Surgitech (Brisbane, Australia). Commercial silicon covered nitinol oesophageal stents (Cook Medical, Bloomington, IN, USA) were kindly gifted by Professor Rajvinder Singh, Director of Gastroenterology at the Lyell McEwin and Modbury Hospitals, South Australia. Chronosil AL 80A 5% Silicone was purchased from AdvanSource Biomaterials (USA). DTX was purchased from Jinhe Bio-Technology (Shanghai, China). Acetonitrile (HPLC grade) and Tween-80 were purchased from Merck (Sydney, Australia). Analytical grade tetrahydrofuran (THF), acetic acid, sodium hydroxide and ammonium acetate were purchased from Chem-Supply (Adelaide, Australia).

The human oesophageal squamous carcinoma cell line (KYSE-30) was purchased from China Cell Bank (Shanghai, China). RPMI-1640 medium was obtained from Gibco (Shanghai, China). Fetal bovine serum (FBS) was obtained from Honbiotech (Jinan, China) and streptomycin, 3-(4,5-Dimethylthiazol-2-yl)-2,5-diphenyltetrazolium bromide (MTT), Annexin V-FITC/PI apoptosis detection kit, primary antibody diluents, enhanced chemiluminescence (ECL) reagent, penicillin (100 units/mL) and streptomycin (100 µg/mL) were purchased from Dalian Meilun Biotechnology (Dalian, China). Dimethyl sulfoxide (DMSO) was purchased from Sigma (USA). Hoechst 33,342, 6× loading buffer, low efficiency radioimmunoprecipitation assay (RIPA) buffer and phenylmethanesulfonyl fluoride (PMSF) were purchased from Beyotime Biotechnology (Shanghai, China). Polyvinylidene fluoride (PVDF) membrane was purchased from Millipore (Billerica, MA, USA). Human B-cell lymphoma 2 (Bcl-2), Bax antibody, poly[adenosine diphosphate-ribose] polymerase (PARP) antibody were purchased from Proteintech (Wuhan, China). Anti-β-actin was obtained from ZS Bio (Beijing, China), glyceraldehyde-3-phosphate dehydrogenase (GADPH) antibody was obtained from Affinity Biosciences (Cincinnati, OH, USA), goat antimouse and goat antirabbit IgG were purchased from Origene (Beijing, China).

### 3.2. Fabrication of Docetaxel-Loaded Nitinol Stents

DTX-eluting coatings were prepared on stents via dip-coating with solutions of DTX and Chronosil in THF. Chronosil (24 g) was dissolved in THF in a 200 mL volumetric flask with heating at 40 °C for 18 h to afford a 12% *w*/*v* solution. DTX (470 or 690 mg) was dissolved in THF (10 mL) and then added to separate Chronosil solutions (200 mL) with sonication to afford solutions consisting of 1.92% *w*/*w* and 2.79% *w*/*w* of DTX relative to Chronosil, respectively.

The stents were carefully rinsed with ethanol and then distilled water and dried in an oven (60 °C) for 12 h. The stents were suspended on the dip-coating frame (SI, Figure S3) of a dip-coating machine (Model TL0.01, MTI Corporation, Richmond, CA, USA), and lowered into a beaker containing the DTX/Chronosil solution (200 mL). After 1 min the stent was retracted at a speed of 6.5 mm/s and allowed to air-dry for 10 min before repeating the dip-coating process. The procedure was repeated twice to afford thicker coatings. The DTX-Chronosil coated stents were dried in an oven (60 °C) for 48 h. Additionally, Chronosil coated stents without DTX (drug-free control) were fabricated using the same method.

### 3.3. Scanning Electron Microscopy (SEM)

SEM was conducted on a Zeiss Merlin Field Emission Gun Scanning Electron Microscope (Jena, Germany). The drug-free and DTX-loaded stents were cut into small pieces (5 × 5 mm), mounted on the sample holder and sputter-coated with platinum (5 nm thickness). Photomicrographs were recorded at an accelerating voltage of 2 kV at 1000 magnification.

### 3.4. Mechanical Properties

Tensile mechanical properties of commercial silicon covered nitinol oesophageal stents and DTX-loaded stents were recorded on a Shimadzu, EZ-LX mechanical tester at a speed of 300 mm/min and using a 500 N load cell. The tensile strength was measured for all stents until the diameter of the stent was reduced to 8 mm. The results are reported as the average of three replicates.

Stent introducer test: A commercial stent introducer (Evolution^®^, Cook Medical, Bloomington, IN, USA) was used to demonstrate the flexibility and recoverability of the drug-loaded stents. After the drug-loaded stent was compressed into the catheter, it was then immediately released, and the process of compressing and expanding was repeated at least ten times. The appearance of the stent was visually examined.

### 3.5. Drug Extraction and HPLC Determination of DTX

Drug-loaded stents were cut into small pieces (30.95 ± 0.15 mg) using a punch, accurately weighed (*n* = 3) and then the polymer coating and DTX were dissolved in THF (1 mL). The solution was then added to 9 mL mixture of ammonium acetate buffer (0.02 M, 25 mL, pH 5) and acetonitrile (43:57% *v*/*v*). The samples were sonicated for 30 min, filtered through a 0.45 µm polyvinylidene difluoride (PVDF) syringe filter and the DTX concentration was determined via HPLC. Examples of calibration curves and DTX chromatograms are provided in the SI (Figure S4).

HPLC was conducted using a method described by Shaikh et al. [17] on a Shimadzu system consisting of a DGU-20AS degasser, SIL-20A HT autosampler, LC-20AD pump unit and a SPD-M20A UV–vis detector (λ = 230 nm). The system was fitted with an Agilent Eclipse XDB C18 column (4.6 × 150 mm, 3.5 μm particle size) and the oven temperature was set to 25 °C. The mobile phase consisted of ammonium acetate buffer (0.02 M, pH 5) and acetonitrile (43:57% *v*/*v*) at a flow rate of 1 mL/min, and the injection volume was 20 μL.

### 3.6. In Vitro Drug Release

The in vitro drug release studies were performed in release media containing 0.1 M phosphate buffer with 0.1% *v*/*v* Tween 80 (pH 6.5). The DTX-loaded stents were placed in 200 mL of the release media and maintained at 37 °C and 175 rpm on an orbital incubator. Periodically, the release media were collected and completely replaced with fresh media to maintain sink condition. The amount of DTX release was determined via HPLC (vide supra). The in vitro release studies were performed in triplicate.

### 3.7. Stability Studies

#### 3.7.1. Accelerated Stability Studies

DTX-loaded stents were cut into small pieces (30 ± 1 mg), accurately weighted and sealed in separate aluminum foil bags. The samples were stored at either 25 °C/65% relative humidity (RH), 40 °C/75% RH or 25 °C for 3 months. The physical appearance was observed after storage, and the chemical stability of DTX in the stent pieces was analysed via the HPLC (vide supra).

#### 3.7.2. Stability of DTX in Drug-Loaded Stents to Gamma and Ultraviolet (UVC) Irradiation

The DTX-loaded stents were sterilized using gamma or ultraviolet (UVC) irradiation as described by Shaikh et al. [17]. Gamma irradiation (25 kGy) was performed on DTX-loaded stent pieces (20.8 ± 0.1 mg) at room temperature using a ⁶⁰Co irradiator at Steriteck (Australia). UV irradiation was conducted by inserting the stents in a chamber equipped with an Osram 15 W UV light (λ = 254 nm) for 20 min. Subsequently, the DTX was extracted from the stents and quantified via HPLC (vide supra).

### 3.8. Cell Culture Maintenance

Human oesophageal squamous carcinoma (KYSE-30) cell line was cultured in RPMI-1640 medium supplemented with 10% *v*/*v* heat-inactivated FBS, penicillin (100 units/mL) and streptomycin (100 µg/mL). Cells were maintained in a 37 °C humidified incubator with 5% CO_2_.

#### 3.8.1. Cell Proliferation Assay

To examine the effect of the formulations (pure DTX and DTX released from the DESs) on cell viability, a colorimetric MTT assay were conducted in triplicate. KYSE-30 cells were seeded at a density of 2 × 10⁴ cells/well (200 µL/well) in 96-well plates and incubated at 37 °C in 5% CO_2_ atmosphere for 12 h. Different concentrations of DTX in DMSO (0.1, 0.5, 1, 5 and 10 µg/mL) were prepared and cells were treated for 24, 48 and 72 h. For stent formulations, the drug-loaded stents were cut into small pieces (22.4 mg ± 0.26) and soaked individually RPMI-1640 medium (1 mL) for 24 h. The stents then were removed from the solutions and the supernatants were collected. The amount of DTX released from DESs with 1.92 and 2.79% *w*/*w* loadings in 1 mL 1640 medium for 24 h was found to be 9.27 µg and 20.37 µg, respectively. To compare the effect of the DTX released from the DESs on tumour cells at lower concentrations, samples were diluted with more medium to afford drug concentrations of 0.01, 0.1, 0.25, 0.5, 1, 5 µg/mL. Cells were then treated and after 48 h, MTT stock solution (20 μL, 5 mg/mL) was added to each well and incubated at 37 °C for 4 h. MTT was then removed and 150 μL of DMSO added to each well to dissolve formazan crystals. Absorbances were then measured on a Thermo Multiskan GO microplate reader (Thermo-1510, Waltham, MA, USA) at an optical density of 570 nm. Cell viability was then expressed as a percentage compared with the control group (untreated cells).

#### 3.8.2. Hoechst Assay

To visualize the effect of pure DTX and DTX released from the DESs on the KYSE30 cells’ nuclei, Hoechst assay was conducted. KYSE30 cells were seeded at a density of 5 × 10⁴ cells/well (1 mL/well) in 24-well plates and incubated at 37 °C in 5% CO_2_ atmosphere for 12 h. Cells were then exposed to pure DTX solution (1 µg/mL) or DTX released from DESs (1 µg/mL) for 48 h. The treated cells were then fixed with methanol/acetic acid solution (3:1 *v*/*v*) for 10 min and stained with Hoechst 33,342 (10 µg/mL) for 10 min. The cells were then visualized with a fluorescence microscope (Excitation/emission at 340/460 nm, TE2000-S; Nikon, Tokyo, Japan).

#### 3.8.3. Apoptosis Assay

To quantify cytotoxicity effects, KYSE-30 cells were seeded in 6-well plates at a density of 2 × 10^5^ cells/well (2 mL/well) and incubated for 24 h. Following treatment with different concentrations of pure DTX or DTX released from the DESs (1 µg/mL), cells apoptosis was then measured by cell surface phosphatidylserine in apoptotic cells using an Annexin V-FITC/PI apoptosis detection kit and analyzed by fluorescence-activated cell sorting (FACS) flow cytometry (Becton Dickinson, Franklin Lakes, NJ, USA) with emission filters of 525 and 575 nm.

#### 3.8.4. Western Blotting Assay

Cell protein extraction: KYSE30 cells were plated at a density of 2 × 10^5^ cells/well (2 mL/well) and incubated at 37 °C in 5% CO_2_ atmosphere for 12 h. Cells were then treated for 48 h with pure DTX solution (1 µg/mL) or DTX released from the DESs (1 µg/mL). Following incubation, the media were removed, and the cells were washed twice with PBS and lysed by 100 µL buffer (low-efficiency radioimmunoprecipitation assay (RIPA) buffer: phenylmethanesulfonyl fluoride (PMSF) 99:1 (*v*/*v*)). The cells were then scraped from the wells with a clean spatula, transferred to a microcentrifuge tube, and placed on ice for lysis for 30 min. The lysed cells were then collected via centrifugation (1400 rpm, 15 min) at 4 °C. The supernatant was then transferred into a clean microcentrifuge tube; to determine protein concentration, an aliquot (4 µL) was combined with the AB solution (A solution: B solution = 50:1 (*v*/*v*), 200 µL) as per BCA kit instructions (Thermo Fisher Scientific, Waltham, CA, USA). Protein concentration was measured at a wavelength of 570 nm, and the provided standard protein was used as a control for concentration calibration. Subsequently, 1/5 of the total volume of 6× protein loading buffer was added, inactivated at 100 °C for 5 min and stored at −20 °C.

Western blotting: Proteins were separated by sodium dodecyl sulfate-polyacrylamide gel electrophoresis (SDS-PAGE) and transferred onto a polyvinylidene fluoride (PVDF) membrane. The membranes were washed and blocked with 20 mM Tris-buffered saline and 0.1% Tween-20 (TBST) buffer containing 5% *w*/*v* nonfat dry milk overnight before being incubated with antibodies against human Bcl-2, Bax, PARP and β-actin. GADPH and β-actin antibodies were used as a loading control to correct the loaded protein amount. All primary antibodies were diluted by 1:1000 in primary antibody diluents according to the assay instructions. The secondary antibodies used were either goat antimouse or goat antirabbit IgG, depending on the primary antibody used. Antibody binding was detected by enhanced chemiluminescence reagent according to the assay instructions and quantified by densitometry using a ChemiDoc XRS+ System Bio-Rad, Hercules, CA, USA).

## 4. Conclusions

A drug-loaded stent was developed for the first-time using dip coating technique for localized administration of DTX to treat oesophageal cancer. Initially, DTX loaded polyurethane solution was prepared and the bare nitinol stent was dip-coated in the solution. The mechanical studies performed on DTX-loaded stents showed lower ultimate tensile strength, toughness and Young’s modulus when compared to commercial stents. Additionally, no peeling or fracture was observed on the coat’s surface after compressing or releasing using the stent introducer. The stents displayed a sustained release profile of DTX over 33 d and reasonable stability to gamma irradiation was achieved, although accelerated storage at elevated temperatures (40 °C) resulted in significant DTX degradation. The cell viability assays confirmed that the released DTX from the dip-coated stents had similar anticancer activity in vitro compared to pure DTX against oesophageal cancer cells. Therefore, this study has shown that the dip-coated stent has the potential to be considered a platform for DTX delivery to oesophageal tumours, although more studies are required in the future for optimizing the mechanical properties, stability and animal studies.

## Figures and Tables

**Table 1:** Mechanical properties of commercial fully covered, and 1.92% *w*/*w* and 2.79% *w*/*w* DTX-loaded oesophageal stents (*n* = 3).

Sample	Ultimate Tensile Strength (KPa)	Elongation at 8 mm Diameter (%)	Toughness (J m^−3^)	Young’s Modulus (kPa)
1.92% *w*/*w* DTX Loaded stent	728 ± 87.0	167 ± 1.53	34.2 ± 1.38	2.41 ± 0.16
2.79% *w*/*w* DTX Loaded stent	543 ± 69.6	154 ± 11.0	27.0 ± 5.68	2.05 ± 0.34
Commercial stent	2135 ± 281	143 ± 5.05	62.6 ± 6.89	4.35 ± 0.17

**Table 2:** Remaining DTX in stent pieces after storage under various conditions over a period of 3 months (*n* = 3).

DTX Remaining (%) after Certain Storage Times and Conditions				
DTX-Loaded Stent	Storage Condition	1 month	2 months	3 months
1.92% *w*/*w*	25 °C	95.0 ± 1.40	96.7 ± 10.6	90.0 ± 1.08
	25 °C/60% RH	95.8 ± 3.27	97.9 ± 6.49	94.6 ± 5.72
	40 °C/75%RH	96.7 ± 2.43	78.7 ± 23.1	73.2 ± 17.0
2.79% *w*/*w*	25 °C	96.8 ± 5.01	91.64 ± 5.42	90.0 ± 3.07
	25 °C/60% RH	97.3 ± 3.26	97.6 ± 5.42	93.5 ± 8.21
	40 °C/75%RH	95.2 ± 2.92	83.8 ± 10.0	81.7 ± 6.57

**Table 3:** Effect of gamma and UV irradiation on drug content of DTX loaded stents.

Sample	Weight of Sample (mg)	Drug Content (µg)	% DTX Remaining
Before UV/γ irradiation	15.3 ± 0.14	158.3 ± 6.13	100
After UV irradiation	15.2 ± 0.24	101.1 ± 0.50	63.9
After γ irradiation	15.6 ± 0.11	145.5 ± 2.52	92.0

## Data Availability

Not applicable.

## Supplementary Materials

The following are available online at https://www.mdpi.com/article/10.3390/ph14040311/s1, Table S1: Stents diameters, before and after the coating. Figure S1. The series of images from left to right show the deployment of the dip-coated stent using a stent introducer and the intact stent after deployment. Figure S2. DESs and pure DTX induced cell apoptosis in KYSE30 cells via Annexin V-FITC/PI double staining. Apoptosis rate of KYSE30 cells after treatment with pure DTX (1 μg/mL) or DESs solution for 48 h were detected by flow cytometric analysis after Annexin V-FITC/PI double staining. Figure S3: Process of dip coating of metal nitinol oesophageal stent into the DTX-Chronosil solution. Figure S4. (A) Standard calibration curve of docetaxel (in mobile phase medium (mixture of ammonium acetate buffer (0.02 M, 25 mL, pH 5) and acetonitrile (43:57% v/v)) by HPLC. (B) HPLC chromatogram of docetaxel in mobile phase medium. DTX retention time: 4.324 min.
