# Supplementary material for: Drug-Loaded, Polyurethane Coated Nitinol Stents for the Controlled Release of Docetaxel for the Treatment of Oesophageal Cancer
Source: Pharmaceuticals (Basel). 2021 Apr 1;14(4):311. doi: 10.3390/ph14040311 (PMC8067330; doi:10.3390/ph14040311)
Supplement: Supplementary file 1 [file pharmaceuticals-14-00311-s001.pdf]

Supplementary information

Drug-loaded, polyurethane coated nitinol stents for the controlled release of docetaxel for the treatment of oesophageal cancer

Paris Fouladian [1], Qiuyang Jin [2], Mohammad Arafat [1], Yunmei Song [1], Xiuli Guo [2,*], Anton Blencowe [3,*] and Sanjay Garg [1,*]

[1] Pharmaceutical Innovation and Development (PIDG) Group, Clinical and Health Sciences, University of South Australia, Adelaide, SA 5000, Australia; paris.fouladian@mymail.unisa.edu.au (P.F.); mohammad.arafat@mymail.unisa.edu.au (M.A.); May.Song@unisa.edu.au (Y.S.)

[2] Key Laboratory of Chemical Biology (Ministry of Education), Department of Pharmacology, School of Pharmaceutical Sciences, Shandong University, Jinan 250012, China; jcas09@163.com

[3] Applied Chemistry and Translational Biomaterials (ACTB) Group, Clinical and Health Sciences, University of South Australia, Adelaide, SA 5000, Australia

* Correspondence: guoxl@sdu.edu.cn (X.G.); anton.blencowe@unisa.edu.au (A.B.); sanjay.garg@unisa.edu.au (S.G.)

Table S1. Stents diameters, before and after the coating.

Stent	Before coating (mm)	After coating (mm)
Flange length	10.01 ± 0.03	10.07 ± 0.11
Flange outer diameter	30.35 ± 0.58	31.12 ± 0.12
Cylinder (central area) length	40.04 ± 0.04	40.23 ± 0.06
Cylinder (central area) outer diameter	28.03 ± 0.05	28.63 ± 0.52
Wall thickness (including the metal mesh)	0.41 ± 0.03	0.46 ± 0.01

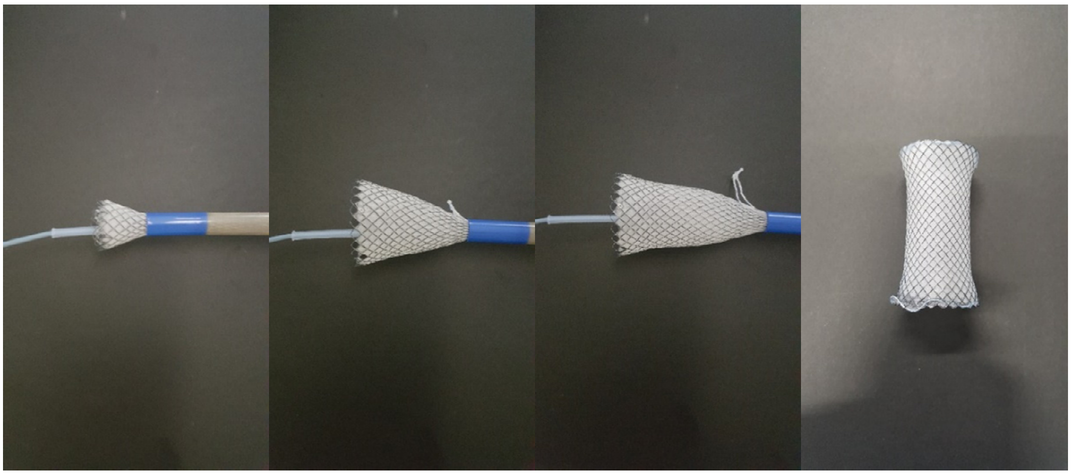

Figure S1. The series of images from left to right show the depolyment of the dip-coated stent using a stent introducer and the intact stent after deployment.

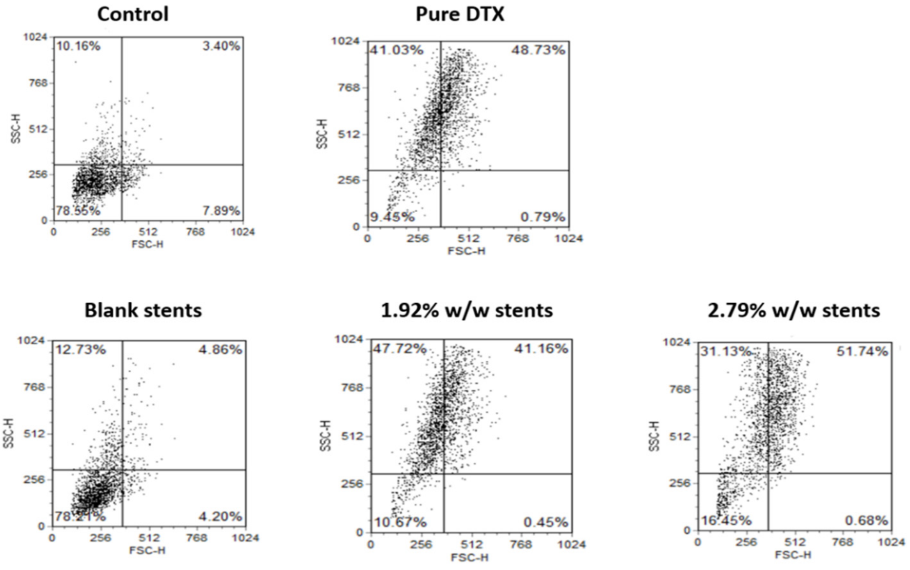

Figure S2. DESs and pure DTX induced cell apoptosis in KYSE30 cells *via* Annexin V-FITC/PI double staining. Apoptosis rate of KYSE30 cells after treatment with pure DTX (1 g/ml) or DESs solution for 48 h were detected by flow cytometric analysis after Annexin V-FITC/PI double staining.

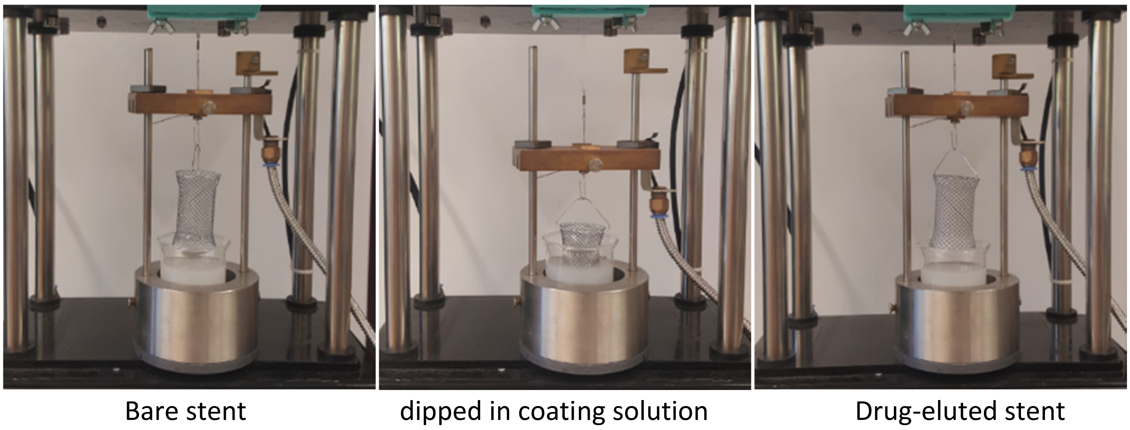

Figure S3. Process of dip coating of metal nitinol oesophageal stent into the DTX-Chronosil solution.

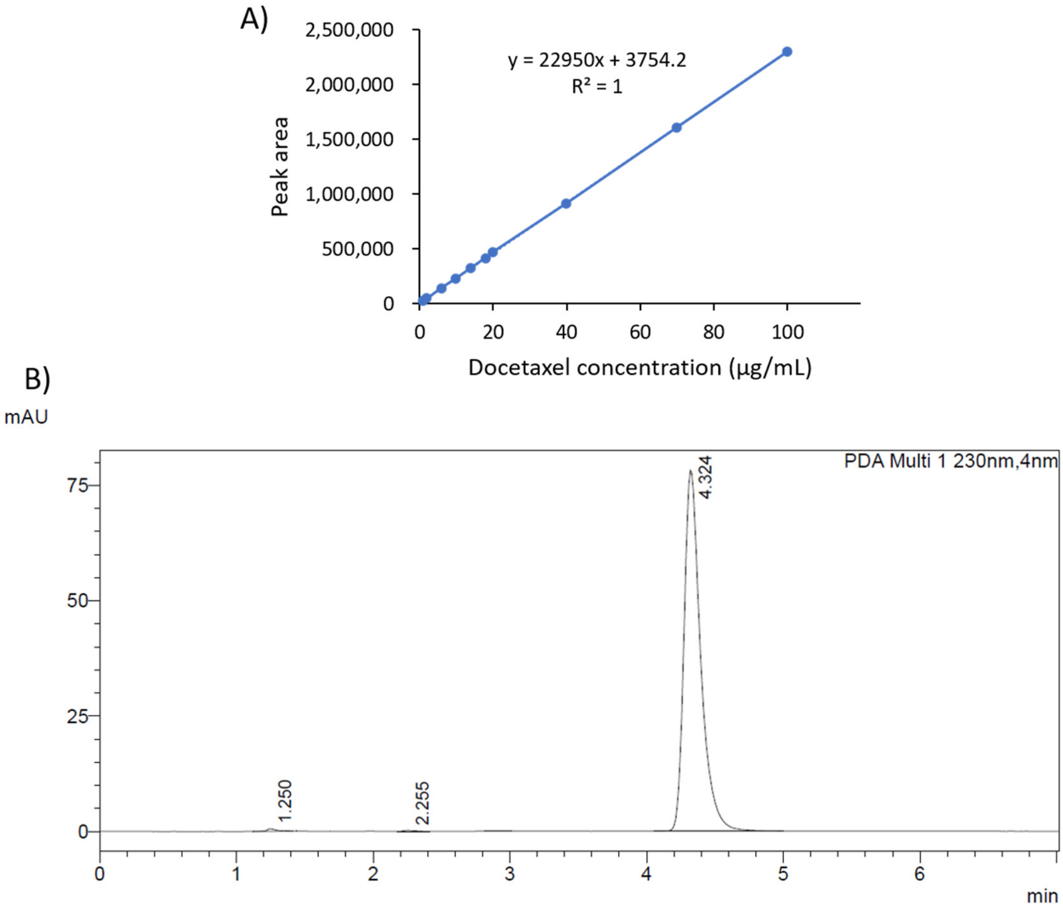

Figure S4. A) Standard calibration curve of docetaxel (in mobile phase medium (mixture of ammonium acetate buffer (0.02 M, pH 5) and acetonitrile (43:57% v/v)) by HPLC. B) HPLC chromatogram of docetaxel in mobile phase medium. DTX retention time: 4.324 min
